# Chronic low back pain patients’ use of, level of knowledge of and perceived benefits of complementary medicine: a cross-sectional study at an academic pain center

**DOI:** 10.1186/s12906-017-1708-1

**Published:** 2017-04-04

**Authors:** Julie Dubois, Emmanuelle Scala, Mohamed Faouzi, Isabelle Decosterd, Bernard Burnand, Pierre-Yves Rodondi

**Affiliations:** 1 Institute of Social and Preventive Medicine (IUMSP), Lausanne University Hospital, Route de la Corniche 10, 1010 Lausanne, Switzerland; 2 Pain Center, Department of Anesthesiology, Lausanne University Hospital, Rue du Bugnon 46, 1011 Lausanne, Switzerland

**Keywords:** Chronic pain, Back pain, Complementary medicine, Communication

## Abstract

**Background:**

Chronic pain patients often use complementary medicine (CM) to alleviate their pain; however, little is known about the use of CM by chronic low back pain (cLBP) patients. We investigated the frequency of use of CM by cLBP patients, the perceived effects of these therapies, patients’ knowledge regarding CM, and patient-physician communication regarding CM.

**Method:**

A cross-sectional survey was conducted from November 2014 to February 2015. A questionnaire was distributed by physicians to 238 consecutive patients consulting for cLBP at the Pain Center of Lausanne University Hospital, Switzerland. Poisson regression model was used to analyze patients’ level of knowledge regarding various CMs, and the logistic regression model was used to assess CM use for cLBP.

**Results:**

The questionnaire was returned by 168 cLBP patients (response rate: 70.6%). Lifetime prevalence of CM use for cLBP was 77.3%. The most commonly used therapies were osteopathy (48.8%), massage (45.2%) and acupuncture (31.6%), rated for their usefulness on a 0–10 scale as a mean ± SD of 5.4 ± 2.7, 5.9 ± 2.5 and 3.8 ± 3.2, respectively. The CM treatment best known by patients was osteopathy, followed by massage and acupuncture. If their doctors proposed CM as a treatment for cLBP, 78% of participants reported being very or somewhat likely to try CM. Respondents with CM health insurance were more likely to use CM (OR = 2.26; 95%CI: 1.07–4.78; *p* = 0.031) for cLBP. Respondents having experienced cLBP for more than five years were more likely to use CM to treat their cLBP than respondents having experienced cLBP for one year or less (OR = 2.84; 95%CI: 1.02–7.88; *p* = 0.044).

**Conclusions:**

More than three-quarters of cLBP patients in our sample did use CM to treat their cLBP. The results showed that the most commonly used therapies were not necessarily the highest rated in terms of perceived usefulness. These results highlight the importance of developing integrative pain centers in which patients may obtain advice regarding CM treatments.

**Electronic supplementary material:**

The online version of this article (doi:10.1186/s12906-017-1708-1) contains supplementary material, which is available to authorized users.

## Background

In Western countries, low back pain is one of the most common patient complaints*.* In the US, the annual prevalence of back pain ranges from 15 to 45% [1], and back pain is the second most common reason for ambulatory care visits [2]. In Europe, the global prevalence of low back pain was estimated to be 13% for males and 10% for women in 2010 [3]. In Switzerland, 47% of women and 39% of men suffered from diverse back problems during the four weeks preceding a survey conducted in 2007 [4]. According to Wieser [5], “low back pain (LBP) is a leading cause of reduced work performance and disability” in Switzerland. Low back pain is known to be at high risk of becoming chronic [6]. The overall prevalence of chronic pain in the European population is estimated at 19%, and nearly half of those patients suffer from chronic back pain [7]. A study conducted in North Carolina (US) showed a significant increase in the prevalence of chronic low back pain (cLBP) over a 14-year period, from 3.9% in 1992 to 10.2% in 2006 [8].

Because chronic pain is difficult to manage, patients often turn to complementary medicine (CM) to treat or alleviate their discomfort [9]. In the case of chronic low back pain, a systematic review showed that most pharmacological treatments had only a limited impact on pain relief and function improvement [10]. This, and the side effects pharmacological treatments may have, could be a reason why cLBP patients chose to try complementary medicine therapies [11, 12]. CM use could also be linked to cLBP patients seeking an active role in their treatments and thus turning to CM as a complement to conventional treatments [9, 12]. In the US, 52% of patients with chronic pain claimed to use or have used CM [9], and back pain is the most commonly reported condition in the use of CM [13]. One US study evaluating CM use among cLBP patients revealed that most patients had used chiropractic (45%) or massage (21%) for their back pain [14]. Some CM therapies, such as acupuncture, have been shown to be moderately effective in the treatment of low back pain [10, 15, 16]. In Switzerland, 25% of the population aged 15 or older had used at least one type of CM during the previous 12 months [17], and CM users primarily suffered from migraines, arthritis and allergies [18]; no survey included specific questions regarding the use of CM for back pain.

This study has several objectives: to investigate the prevalence of CM use in patients visiting an academic pain center in Switzerland, to examine patients’ perceptions of the effects of these therapies, to explore their knowledge of CM, and to investigate patient-physician communication regarding CM. A secondary objective is identifying factors associated with CM use for cLBP.

## Methods

### Study design

This cross-sectional study occurred from November 2014 to February 2015. Questionnaires were distributed by physicians during their encounters with every patient consulting for cLBP at the Pain Center at the Lausanne University Hospital in the French speaking part of Switzerland.

### Setting and participants

Recruitment of participants for the study was conducted within the pain center at the Lausanne University Hospital. For a period of 8 weeks, at the end of each encounter with a cLBP patient, physicians gave the patient a printed questionnaire with a stamped envelope including the return address. Thus, questionnaires were given to all consecutive eligible cLBP patients during the time period of the study. At the beginning of the questionnaire, the goal of the study was explained to the participants, confidentiality was confirmed, and specific terms such as chronic LBP and the CM definition used for this study were explained. Patients were required to return the questionnaire within 3 weeks.

Inclusion criteria were patients consulting at our pain center for cLBP lasting longer than six months and who spoke fluent French. Our study considered pain to be chronic after six months, consistent with the definition of the American Academy of Pain Management [19]. Patients under 18, unable to understand written or verbal instructions to complete the questionnaire or considered unable to answer the questionnaire by medical or nursing staff (for example, suffering from serious psychiatric disease) were excluded.

It is worthy to note that the pain center were the study took place offered only conventional treatments at the time of the study, thus CM use could not have been increased due to a CM offer at the pain center.

### Variables

We developed a 25-item questionnaire that was divided into three sections. The first section (seven items) explored socio-demographic data. The second section (five items) sought to describe the duration and burden of cLBP. The last section (13 items) inquired about the patient’s knowledge, use of and perceptions regarding CM in the context of cLBP (see Additional file 1).

Self-reported knowledge regarding CM was evaluated with an item consisting of 21 sub-items assessing the 21 different CM techniques presented in the questionnaire. For each technique, knowledge could reach a maximum of 3 points (from 0 points for “I do not know that technique” to 3 points for “I know this technique very well”). The knowledge score was the sum of the 21 items, resulting in a 63-point maximum score for the entire set of sub-items.

### Data sources

The questionnaire combined results from the literature search and discussions within our team but was primarily based on a previously published instrument [14]. We also adapted some of our questions from the American National Health Interview Survey (NHIS) [20]. The questionnaire was subjected to cognitive testing [21]. The goal was to determine how respondents interpreted the intent and meaning of survey questions and whether these responses matched the interpretations of the researchers. Cognitive interviews were conducted with ten volunteer cLBP patients and with ten healthy volunteers from the general public, all with a range of various socio-demographic characteristics [20]. The resulting modifications were conducted to enhance patients’ comprehension of the questions. The protocol and the questionnaire were approved by the ethics committee of the Canton of Vaud.

### Statistical analysis

Data quality and completeness (unusual values, consistency, missing values) were checked to ensure data quality. Categorical data were summarized by frequency and percentage and the mean (±SD) for the age. Univariable logistic regression analyses were performed to identify factors associated with CM use for cLBP. The strength of the association was measured by the OR (Odds Ratio) and *p*-values. Knowledge regarding various CMs was assessed using the univariable Poisson regression model, and the strength of the association was measured by the IRR (Incidence Rate Ratio) and associated *p*-values. For both outcomes, CM use, and knowledge regarding various CM factors, outcomes at a level of 20% (*p*-value < 0.20) were considered in a backward stepwise procedure to fit a multivariable model. Statistical analysis was conducted using Stata 14 software (StataCorp. 2015. Stata Statistical Software: Release 14. College Station, TX: StataCorp LP).

## Results

Of the 238 eligible patients, 168 (response rate: 70.6%) completed the questionnaire, 9 declined to participate and 61 did not return the questionnaire. Socio-demographic characteristics are described in Table 1.

**Table 1:** Patients’ socio-demographic characteristics (*N* = 168)

Age	
Mean (±SD)	60 (±16.4)
Sex	
Male	71 (42.8)
Female	95 (57.2)
Origin	
Swiss	113 (69.3)
Other	50 (30.7)
Educational level	
Basic/apprenticeship	88 (59.1)
Professional diploma/high school/college	33 (22.1)
University	28 (18.8)
Marital status	
Single	23 (13.7)
Married/civil partnership	90 (53.9)
Divorced/separated	38 (22.8)
Widower	16 (9.6%)
Complementary medicine health insurance	
Yes	98 (60.1)
No	58 (35.6)
Does not know	7 (4.3)
Duration of pain	
1–12 months	23 (14.3)
1–5 years	62 (38.5)
More than 5 years	76 (47.2)

Results are expressed in number of participants (percentage)

Approximately half (46.9%) of the participants had experienced cLBP for more than 5 years and 39% for 1 to 5 years. Eighty-three percent had experienced LBP every day or nearly every day in the previous 6 months. On a 0 to 10 numeric-rating scale on which 0 indicated “not bothering at all” and 10 indicated “extremely bothering”, level of discomfort was rated as a mean ± SD of 7.3 ± 1.9.

CM had been used by 77.3% of the participants to specifically treat cLBP, with a median of 2 methods per person used (IQR: 4). Among cLBP CM users, the most common therapies used were osteopathy (48.8%), massage (45.2%) and acupuncture (31.6%). Therapies used and their perceived usefulness, rated on a 0 to 10 numeric rating scale (0 being useless and 10 being extremely helpful), are presented in Table 2. There was little variation in the perceived usefulness of the different therapies, with results ranging from 3.5 to 5.9.

**Table 2:** Complementary medicine used and its rated usefulness against chronic low back pain

CM therapies	Percentage of users	Perceived usefulness (mean ± SD)^a^
Osteopathy	48.8	5.4 ± 2.7
Therapeutic massage	45.2	5.9 ± 2.5
Acupuncture	31.6	3.8 ± 3.2
Other	21.5	
Homeopathy	14.9	3.83 ± 2.5
Reflexology	14.9	4.0 ± 2.6
Aromatherapy	10.7	4.2 ± 2.8
Kinesiology	9.5	5.1 ± 3.0
Meditation	8.9	4.8 ± 2.8
Herbal medicine	8.9	4.3 ± 2.8
Reiki	8.9	4.5 ± 3.9
Yoga	8.3	3.5 ± 2.9
Magnetism	6.0	4.7 ± 3.6
Chinese herbs	5.4	3.9 ± 3.6
Hypnosis	4.8	5.1 ± 3.8
Shiatsu	4.8	3.5 ± 3.3
Sophrology	4.8	3.9 ± 2.5

^a^Usefulness was rated on a 0 to 10 scale (0 being useless and 10 being extremely helpful)

Concerning knowledge of CM therapies, the mean ± SD score for knowledge of the 21 CM types proposed in the questionnaire was 12.1 ± 9.8 points of 63 points. Specifically, the mean scores were 9 points (SD = 7.1) for men and 15 points (SD = 11) for women. The best-known method was osteopathy, followed by massage and acupuncture. Women were more likely to have a higher score than men. Most participants obtained information about CM through family and friends (62.5%), followed by physicians (42.3%) and the media (25.6%). Adjusted IRR from multivariable analysis are shown in Table 3. Compared with respondents aged 21–48, respondents aged 49–59 had a better knowledge of CM. Being a woman, of Swiss nationality, and having health insurance that covered CM were also predictors of greater knowledge of CM.

**Table 3:** Poisson regression analysis of patients’ knowledge regarding complementary medicine and associated factors

Variables	Univariable analysis		Multivariable analysis	
	IRR (95% CI)	*p*-value	IRR (95% CI)	*p*-value
Sex				
Female	1.61 (1.45–1.79)	<0.001	1.57 (1.40–1.75)	<0.001
Male	1 ref	-	1 ref	-
Age				
21–48	ref	-	ref	-
49–59	1.23 (1.09–1.39)	0.001	1.19 (1.05–1.35)	0.005
60–74	0.65 (0.56–0.76)	<0.001	0.67 (0.58–0.78)	<0.001
75–88	0.72 (0.62–0.85)	<0.001	0.64 (0.55–0.75)	<0.001
Education				
Basic/apprenticeship	ref	-	-	-
Professional diploma/high school/college	1.06 (0.94–1.20)	0.30	-	-
University	1.03 (0.90–1.18)	0.65	-	-
Marital status				
Single	ref	-	-	-
Married/civil partnership	0.97 (0.84–1.13)	0.76	-	-
Divorced/separated	1.15 (0.98–1.36)	0.07	-	-
Widower	0.95 (0.74–1.21)	0.68	-	-
Nationality				
Other	ref	-	ref	-
Swiss	1.37 (1.21–1.55)	<0.001	1.31 (1.15–1.50)	<0.001
Complementary medicine health insurance				
No	ref	-	ref.	-
Yes	1.19 (1.07–1.32)	0.001	1.12 (1.00–1.25)	0.03

Univariable Poisson regression model and the strength of the association were measured by the IRR (Incidence Rate Ratio) with 95% confidence intervals and their associated *p*-values

Regarding communication, the results indicated that if their physician proposed CM as a treatment for cLBP, 58.3% of the participants would be very likely and 18.6% would be somewhat likely to try CM. Of the participants, 45.2% reported that their physicians at the pain center had asked about using CM, and 16.5% reported that their physicians had proposed including CM (primarily acupuncture) in the treatment strategy. Among patients who used CM at least once for their cLBP, 46.7% reported their CM use to their physicians, and in response, 55.6% of the physicians encouraged their patients to continue; 30.5% of physicians did not make any recommendation. Among the participants who did not discuss using CM with their physicians, the majority (45.6%) said that the lack of communication was “because the physician did not ask me about it”.

As a secondary objective, we identified the factors associated with CM use for cLBP. Respondents having CM health insurance coverage were more likely to use CM. Respondents having experienced cLBP for more than five years were more likely to use CM to treat their cLBP than respondents having experienced cLBP for one year or less. Respondents having a professional diploma or having attended high school/college were more likely than respondents with basic educational levels or apprenticeships to use CM for cLBP. However, it was not possible to fit any multivariable model for CM use for cLBP. Detailed results are shown in Table 4.

**Table 4:** Summary data for users (Yes) compared with non-users (No) of complementary medicine for chronic low back pain

	Complementary medicine use for chronic low back pain			
	YES	NO	OR (95% CI)	(*p*-value)
	(*N* = 126)	(*N* = 37)		
Age (mean ± SD)	59 (**±**16.2)	61 (±16.2)	0.99 (0.96–1.01)	0.41
Sex				
Male	50 (40.3%)	19 (51.4)	ref	
Female	74 (59.7%)	18 (48.6)	1.56 (0.74–3.26)	0.24
Origin				
Other	36 (29.5%)	13 (36.1%)	ref	
Swiss	86 (70.5%)	23 (63.9%)	1.35 (0.61–2.95)	0.45
Educational level				
Basic/apprenticeship	66 (55.5%)	22 (73.3%)	ref	
Professional diploma/high school/college	31 (26%)	2 (6.7%)	5.16 (1.14–23-3)	0.03
University	22 (18.5%)	2 (20%)	1.22 (0.43–3.4)	0.70
Marital status				
Single	19 (15.2%)	3 (8.8%)	ref	
Married/civil partnership	67 (53.6%)	10 (58.8%)	0.50 (0.13–1.87)	0.31
Divorced/separated	29 (23.2%)	8 (23.6%)	0.57 (0.13–2.43)	0.45
Widower	10 (8%)	3 (8.8%)	0.32 (0.06–1.6)	0.16
Complementary medicine health insurance				
No	40 (32.5%)	14 (41.2%)	ref	
Yes	81 (65.9%)	17 (50%)	2.26 (1.07–4.78)	0.03
Does not know	2 (1.6%)	3 (8.8%)		
Pain duration				
1–12 months	14 (11.3%)	9 (24.4%)	ref	
1–5 years	48 (38.7%)	14 (37.8%)	2.2 (0.78-6.15)	0.13
More than 5 years	76 (47.2%)	14 (37.8%)	2.84 (1.02–7.88)	0.04

Associations between explanatory variables and outcomes were assessed using logistic regression model and expressed by the Odds-Ratio (OR), 95% confidence interval and *p*-value

## Discussion

More than three quarters of all participants used CM for cLBP at some point in their lives. In our sample, the typical cLBP patient using CM was a female approximately 60 years old, having a professional diploma or having attended high school/college. This greater use of CM and the profile of a typical CM user were also identified in a study on CM use in chronic headaches and cLBP [12].

The most common therapies used to treat cLBP were osteopathy, massage and acupuncture, followed by homeopathy and reflexology. In addition to chiropractic, those therapies were also the most common among patients identified in two recent studies of CM use for back pain [22, 23]. For cLBP patients more specifically, a study investigated the use of and knowledge regarding five CM therapies (chiropractic, tai-chi, massage, acupuncture and meditation) by cLBP patients in the US [14]. That study’s results indicated that approximately half of participants used chiropractic for cLBP, one-quarter used massage, and approximately one-tenth used acupuncture and meditation. Our study showed higher usage rates for massage, acupuncture and meditation; however, both studies showed a preference for manual techniques and acupuncture. Another study on CM conducted in Austria and Germany on the same type of population indicated that patients mostly used thermotherapy, massage and acupuncture [12]. Our study did not include chiropractic use because chiropractic is considered a medical profession in Switzerland and not a CM therapy.

Our results have shown that the therapies that participants knew best were also the most used therapies. However, the perceived helpfulness of some less used and lesser-known therapies was rated higher than the therapies most commonly used. As described previously, the most used therapies were osteopathy, massage, acupuncture, and homeopathy; however, the highest rated were massage, osteopathy, hypnosis and kinesiology. In a study of five CMs used for cLBP (acupuncture, chiropractic, massage, meditation and tai chi), Sherman et al. [14] observed slightly higher rates of perceived helpfulness than within our study population for massage (6 compared with 7), acupuncture (4 compared with 5) and meditation (5 compared with 5). In a study among the US low back pain adult population, more than half of acupuncture, massage and yoga users considered those therapies as having great benefits [23]. According to the National Center for Complementary and Integrative Health (NCCIH), spinal manipulation, acupuncture and massage may actually be beneficial in treating chronic low back pain [24]. Results of RCTs and meta-analysis also indicated that some CMs such as meditation could be suggested as a treatment option to patients with cLBP [25, 26]. Moreover, the American College of Physicians emitted guidelines for the treatment of cLBP which emphasizes on the use of noninvasive treatments such as yoga, acupuncture and tai-chi [10].

Patients having experienced cLBP for more than five years were 2.8 times more likely to have used CM for that condition than patients having experienced cLBP for one year or less, which may indicate that CM treatments are perceived as a late resort for patients with cLBP. This explanation was also suggested in a study among women with back pain in Australia [27].

Patients having CM health insurance coverage were significantly more likely to use CM for cLBP. This finding is consistent with the fact that in Switzerland, supplemental insurance often covers the cost of various CM treatments that are not reimbursed by the mandatory basic health insurance. Thus, it appears that the costs of CM may affect its use by cLBP patients. In addition, patients more interested in CM or who have had positive CM experiences may choose to acquire complementary coverage to support CM. Sherman [14] also observed that patients would be more likely to try CM if their primary care provider thought the treatment was reasonable and if CM came with no extra cost, or a $10 co-pay, than if the patients had to pay for the entire treatment out of pocket.

Three-quarters of the participants were very or somewhat likely to try CM to treat their cLBP if their physicians suggested that treatment. A first step would most likely be to increase physicians’ knowledge of CM and its effectiveness, particularly because most patients ultimately use CM but perhaps not always to the most useful treatments. As noted elsewhere, physicians mention the need to learn more about CM to adequately address patients’ concerns [28]. This finding highlights the importance of physicians’ obtaining knowledge regarding which CM should be recommended or not recommended in the treatment of cLBP. This statement is reinforced by the fact that nearly half of our participants obtained information on CM through a physician sometime in life and thus rely on them as a source of information on the matter. The guidelines of the American College of Physicians [10], which recommend various CM techniques for the management of cLBP could be an important step for improving physicians’ knowledge of useful CM treatments. Furthermore, a survey conducted among healthcare professionals, including physicians, midwifes and nurses, showed that more than 90% of the respondents considered CM could be helpful in the treatment of chronic pain but that they lacked information to advise their patients [29].

Nearly half of the participants reported that their physicians at the pain center asked if they used CM, and nearly half of the CM users did not spontaneously admit such use to their physicians. A recent study [30] indicated that among women using CM for back pain, only one-third informed their general practitioners after using CM. Overall, few studies investigated physician-patient communication in the context of CM for low back pain [22]. Lack of communication may be linked to physicians’ lack of knowledge of CM [28]. Furthermore, it has been shown that in the context of cLBP, effective patient-professional communication strengthened the therapeutic partnership and helped support patient’s self-management ability [31].

Our study had several limitations. First, the study was monocentric and thus may not reflect CM use by cLBP patients in other pain centers. In addition, cLBP patients who are seen at a pain center constitute a selection of complex refractory cases. However, our primary results, such as the prevalence of CM use, were comparable to other studies [12, 22]. Second, our study focused on knowing the prevalence of CM use since they first experienced cLBP and not the participants’ current use of CM or use in the previous year, for example. Such measurements would potentially have shown less CM use; however, because our population comprised chronic pain patients and sought to determine the perceived effectiveness of the CM treatments, we chose to study their long-term use. Third, the number of participants was relatively low because the study calendar allowed only eight weeks of data collection. This limitation renders our results more difficult to generalize. Fourth, it is possible that the score of perceived helpfulness of some less used and lesser-known therapies would be lower once tried more widely. Further investigations on CM use in the context of cLBP on a larger population sample are necessary. Finally, patients more interested in CM may have participated more readily than patients who had no interest, resulting in potential self-selection and biased results and indicating an exaggerated CM use and knowledge.

## Conclusion

CM was used by a majority of cLBP patients in the pain center surveyed. The results indicated that the most commonly used therapies were not necessarily the highest rated in terms of perceived usefulness.

Our results highlight the importance of accumulating more data on the effectiveness of CM for cLBP. Furthermore, these results underline the need to improve patient-physician communication on the matter of CM use. Integrative pain centers in which patients may obtain advice both on conventional and CM treatments should be developed to provide better support for patients with chronic pain.

## Additional file

Additional file 1: Study questionnaire (in French). (DOCX 67 kb)

## Abbreviations

CM: Complementary medicine
cLBP: Chronic low back pain
NHIS: American National Health Interview Survey
NCCIH: National Center for Complementary and Integrative Health
