# Supplementary material for: Chronic low back pain patients’ use of, level of knowledge of and perceived benefits of complementary medicine: a cross-sectional study at an academic pain center
Source: BMC Complement Altern Med. 2017 Apr 4;17:193. doi: 10.1186/s12906-017-1708-1 (PMC5379639; doi:10.1186/s12906-017-1708-1)
Supplement: Additional file 1: — Study questionnaire (in French). (DOCX 67 kb) [file 12906_2017_1708_MOESM1_ESM.docx]

**Institut universitaire de médecine sociale et préventive (IUMSP)**

**Centre d’antalgie**

**Questionnaire**

**Etude sur l’utilisation des médecines complémentaires par les patients présentant des lombalgies chroniques.**

De nombreux patients souffrant de douleurs chroniques ont recours à des thérapies complémentaires. Nous souhaitons connaître votre opinion sur les médecines complémentaires dans le traitement des lombalgies chroniques.

Définitions utilisées dans le cadre de cette étude :

- **Lombalgie**: douleurs dans la partie basse du dos, pouvant dans certains cas descendre dans la fesse, la jambe ou le pied.
- **Médecines complémentaires** (appelées aussi médecines alternatives, parallèles, douces, etc.) : un large ensemble de pratiques de soins qui ne font pas partie de la tradition académique du pays ou qui ne sont pas systématiquement intégrées dans le système de santé (par exemple : l’acupuncture, l’homéopathie, la phytothérapie, l’ostéopathie, …). Quelques exemples figurent à la question 12. Les médecines complémentaires sont parfois prescrites par des médecins.
- Il faut compter au maximum 10-15 minutes pour remplir le questionnaire.
- Toutes les réponses seront traitées de manière anonyme et ne seront pas transmises au médecin qui vous prend en charge au centre d’antalgie.

Nous vous remercions de répondre aux questions suivantes :

**Vos données socio-démographiques :**

**1.** Année de naissance : ……………

**2.** Sexe :  homme  femme

**3**. Pays de naissance : …………………………………………………………………………….

**4**. Nationalité(s) : ………………………………… ……………………………………………

**5**. Etat civil :

célibataire

marié(e) ou partenariat enregistré

séparé(e)/divorcé(e)/partenariat dissout

veuf(ve)

**6.** Votre plus haut niveau de formation achevé :

école obligatoire  maîtrise fédérale, diplôme professionnel (technicum, etc.)

apprentissage  université, haute école

baccalauréat, maturité  Je ne sais pas/ne souhaite pas répondre

**7.** Êtes-vous au bénéfice d’une assurance complémentaire qui couvre des thérapies de médecine complémentaire :

Oui

Non

Je ne sais pas

**Description de votre douleur :**

**8.** Quand avez-vous ressenti, pour la 1ère fois, des lombalgies qui ont duré plus de deux semaines :

Il y a :  moins d’un mois

1-3 mois

3-6 mois

6 mois-1 an

1-5 ans

plus de 5 ans

Je ne sais pas

Je n’ai jamais eu de douleurs lombaires : dans ce cas, le questionnaire s’arrête ici pour vous, merci de nous le renvoyer.

**9.** Durant les 6 derniers mois, à quelle fréquence avez-vous eu des lombalgies ?

Tous les jours, ou presque tous les jours, dans le 6 derniers mois

Au moins un jour sur deux dans les 6 derniers mois

Moins d’un jour sur deux dans les 6 derniers mois

**10.** Sur une échelle de 0 à 10, où 0 signifie “**aucune gène”** et 10 “**extrêmement gênant”**, à quel point vos lombalgies ont été gênantes pour votre qualité de vie durant les 6 derniers mois, en moyenne (entourer le chiffre qui convient) :

0------1------2------3------4------5------6------7------8------9------10

Je ne sais pas

**11.** Durant les 6 derniers mois, avez-vous pris un/des médicament(s) conventionnel(s) (par exemple, un anti-douleur comme Dafalgan, Panadol, etc., ou un anti-inflammatoire comme Aspirine, Algifor, Irfen, etc.) pour vos douleurs lombaires spécifiquement :

Oui

Non

Je ne sais pas

**Les questions suivantes portent sur votre familiarité avec différents types de traitements en médecine complémentaire :**

**12.** Combien **connaissez-vous** chaque méthode listée ci-dessous (veuillez cocher une case par ligne SVP) :

|  | Je connais **très bien** | Je connais **bien** | Je connais **un peu** | Je ne connais **pas du tout** | Je ne sais pas quoi répondre |
| --- | --- | --- | --- | --- | --- |
| Acupuncture |  |  |  |  |  |
| Aromathérapie/huiles essentielles |  |  |  |  |  |
| Art-thérapie (musicothérapie, etc.) |  |  |  |  |  |
| Hypnose |  |  |  |  |  |
| Homéopathie |  |  |  |  |  |
| Massage thérapeutique |  |  |  |  |  |
| Médecine anthroposophique |  |  |  |  |  |
| Médecine ayurvédique |  |  |  |  |  |
| Herbes chinoises (issues de la médecine chinoise) |  |  |  |  |  |
| Méditation |  |  |  |  |  |
| Ostéopathie |  |  |  |  |  |
| Magnétisme |  |  |  |  |  |
| Shiatsu |  |  |  |  |  |
| Réflexothérapie (réflexologie) |  |  |  |  |  |
| Sophrologie |  |  |  |  |  |
| Thérapie neurale |  |  |  |  |  |
| Tai Chi et/ou Qi Gong |  |  |  |  |  |
| Phytothérapie (thérapie par les plantes) |  |  |  |  |  |
| Yoga |  |  |  |  |  |
| Kinésiologie |  |  |  |  |  |
| Reiki |  |  |  |  |  |

**13.** Si vous connaissez certaines médecines complémentaires, comment en avez-vous été informé (plusieurs réponses possibles) :

Famille/amis/collègues

Médecin

Autre(s) professionnel(s) de la santé (spécifiez) : ……………………………………………

Médias (journal, radio, télévision internet, …)

Livre/CD

Je ne connais aucune médecine complémentaire

Je ne sais pas

Autre(spécifiez) :………………………………………………………………………………

**14.** Au cours de votre vie, avez-vous déjà eu recours à une/des médecine(s) complémentaire(s) pour des problèmes de santé physique ou psychique en général :

Oui

Non

Je ne sais pas

**15.** Avez-vous déjà eu recours à une/des médecine(s) complémentaire(s) pour vos lombalgies spécifiquement:

Oui

Non

Je ne sais pas

**→ Si oui :** Veuillez passer à la page suivante.

**→ Si** **non** : Pouvez-vous nous donner les raisons pour lesquelles vous n’avez jamais eu recours à des médecines complémentaires pour vos lombalgies (plusieurs réponses possibles):

Vous n’en avez jamais entendu parler/ne connaissez pas assez bien ce sujet

Vous n’y avez jamais pensé

Vous n’en avez pas besoin

Vous n’y croyez pas/ça n’a pas d’effet

C’est trop cher

Ces méthodes présentent des dangers

Un professionnel de la santé m’a recommandé de ne pas utiliser ces méthodes

La science médicale n’a pas prouvé leur efficacité

Aucune raison

Je ne sais pas

Pour d’autres raisons (spécifiez) : …………………………………………………

**→**Veuillez passer à la question 18.

Veuillez cocher, dans la liste ci-dessous, la/les méthode(s) que vous avez utilisée(s) pour vos lombalgies spécifiquement en les notant sur une échelle de 0 à 10, où 0 signifie « **pas du tout utile** » et 10 « **extrêmement utile** » (entourer **le** chiffre qui convient à côté de chaque méthode cochée. Si cela variait de visite en visite, répondre combien cela vous a aidé dans l’ensemble.)

Acupuncture ….0-1-2-3-4-5-6-7-8-9-10

Aromathérapie / huiles essentielles .…0-1-2-3-4-5-6-7-8-9-10

Art-thérapie (musicothérapie, etc.) .…0-1-2-3-4-5-6-7-8-9-10

Hypnose .…0-1-2-3-4-5-6-7-8-9-10

Homéopathie .…0-1-2-3-4-5-6-7-8-9-10

Massage thérapeutique .…0-1-2-3-4-5-6-7-8-9-10

Médecine anthroposophique .…0-1-2-3-4-5-6-7-8-9-10

Médecine ayurvédique .…0-1-2-3-4-5-6-7-8-9-10

Herbes chinoises (issues de la médecine chinoise) 0-1-2-3-4-5-6-7-8-9-10

Méditation ….0-1-2-3-4-5-6-7-8-9-10

Ostéopathie ….0-1-2-3-4-5-6-7-8-9-10

Magnétisme ….0-1-2-3-4-5-6-7-8-9-10

Shiatsu ….0-1-2-3-4-5-6-7-8-9-10

Réflexothérapie (Réflexologie) ….0-1-2-3-4-5-6-7-8-9-10

Sophrologie ….0-1-2-3-4-5-6-7-8-9-10

Thérapie neurale ….0-1-2-3-4-5-6-7-8-9-10

Tai Chi et/ou Qi Gong ….0-1-2-3-4-5-6-7-8-9-10

Phytothérapie (thérapie par les plantes) ….0-1-2-3-4-5-6-7-8-9-10

Yoga ….0-1-2-3-4-5-6-7-8-9-10

Kinésiologie ….0-1-2-3-4-5-6-7-8-9-10

Reiki ….0-1-2-3-4-5-6-7-8-9-10

Autre (spécifiez) : ….0-1-2-3-4-5-6-7-8-9-10

Autre (spécifiez) : ….0-1-2-3-4-5-6-7-8-9-10

Autre (spécifiez) : ….0-1-2-3-4-5-6-7-8-9-10

Autre (spécifiez) : ….0-1-2-3-4-5-6-7-8-9-10

Je ne connais pas le nom de la méthode ….0-1-2-3-4-5-6-7-8-9-10

**16.** Est-ce que votre/vos traitement(s) en médecine complémentaire, a/ont eu un effet négatif qui vous a/ont particulièrement gêné :

Oui : Décrivez brièvement cet/ces effet(s) négatif(s) : ______________________________________

____________________________________________________________________________

____________________________________________________________________________

Non → Veuillez passer à la question 18.

Je ne sais pas

**17**. Veuillez cocher la/les méthode(s) qui a/ont eu un effet négatif ou qui vous a/ont particulièrement gêné :

Acupuncture

Aromathérapie / huiles essentielles

Art-thérapie (musicothérapie, etc.)

Hypnose

Homéopathie

Massage thérapeutique

Médecine anthroposophique

Médecine ayurvédique

Herbes chinoises (issues de la médecine chinoise)

Méditation

Ostéopathie

Magnétisme

Shiatsu

Réflexothérapie (Réflexologie)

Sophrologie

Thérapie neurale

Tai Chi et/ou Qi Gong

Phytothérapie (thérapie par les plantes)

Yoga

Kinésiologie

Reiki

Autre (spécifiez) : …………………………………………

Autre (spécifiez) : …………………………………………

Autre (spécifiez) : …………………………………………

Je ne connais pas le nom de la méthode

Je ne sais pas

**18**. Pour vos lombalgies, avez-vous :

|  | Oui | Non | Je ne sais pas |
| --- | --- | --- | --- |
| Subi une opération |  |  |  |
| Subi une infiltration |  |  |  |
| Vu un rhumatologue |  |  |  |
| Vu un neuro-chirurgien |  |  |  |
| Vu un chirurgien orthopédique |  |  |  |
| Consulté un centre de la douleur |  |  |  |
| Eu de la physiothérapie |  |  |  |

**19.** Si votre médecin vous proposait une méthode de médecine complémentaire pour vos douleurs lombaires, seriez-vous prêt à essayer :

Pas du tout

Peu probable

Neutre/Pas sûr(e)

Assez probable

Très probable

Je ne sais pas

**20.** Est-ce que votre médecin du CHUV vous a demandé si vous utilisiez une/des médecine(s) complémentaire(s) ? (si vous êtes suivi par plusieurs médecins au CHUV, considérez le médecin que vous avez le plus consulté) :

Oui

Non

Je ne sais pas

**21.** Est-ce que votre médecin du CHUV vous a proposé d’utiliser une/des médecine(s) complémentaire(s) ? (si vous êtes suivi par plusieurs médecins au CHUV, considérez le médecin que vous avez le plus consulté)

Oui (spécifiez laquelle/lesquelles) : ……………………………………………………………………

Non

Je ne sais pas

**22.** Avez-vous dit à votre médecin du CHUV que vous utilisiez une/des médecine(s) complémentaire(s) (si vous êtes suivi par plusieurs médecins au CHUV, considérez le médecin que vous avez le plus consulté pour vos douleurs de dos) :

Oui

Non

Je ne sais pas

Je n’en utilise pas

**→ Si oui**, comment le médecin a-t-il/elle répondu :

Encouragé à continuer le traitement de médecine complémentaire

Demandé d’arrêter le traitement de médecine complémentaire

N’a pas fait de recommandation

N’a pas souhaité en parler

Ne connaissait pas cette/ces thérapie(s)

Je ne sais pas

Autre (spécifiez) :……………………………………………………………

**→ Si non**, pourquoi n’en avez-vous pas parlé :

Parce qu’il/elle ne me l’a pas demandé

Parce qu'il/elle ne comprendrait pas

Parce qu'il/elle ne serait pas content(e)

Parce que j’ai oublié de le mentionner

Parce que cela n’a pas d’importance de le dire

Je ne sais pas

Autre (spécifiez) :…………………………………………………………

**23.** J’ai reçu suffisamment d’informations, de la part du CHUV, sur les **bénéfices** des médecines complémentaires :

Tout à fait d’accord

D’accord

Ni d’accord ni pas d’accord

Pas d’accord

Pas du tout d’accord

Je ne sais pas

Je n'en utilise pas

**24.** J’ai reçu suffisamment d’informations, de la part du CHUV, sur les **risques** des médecines complémentaires :

Tout à fait d’accord

D’accord

Ni d’accord ni pas d’accord

Pas d’accord

Pas du tout d’accord

Je ne sais pas

Je n'en utilise pas

**25**. Avez-vous une/des remarque(s) concernant ce questionnaire : ______________________________________________________________________________________________________________________________________________________________________________________________________________________________________________________________________________________________________________________________________________________________________________________________________________________________________________________________________________________________________________________________

***

Avec nos remerciements pour avoir répondu à ce questionnaire.
